# Development of three multiplex-PCR assays for virulence profiling of different iron acquisition systems in *Escherichia coli*

**DOI:** 10.18502/ijm.v12i4.3930

**Published:** 2020-08

**Authors:** Hamideh Kalateh Rahmani, Gholamreza Hashemi Tabar, Mahdi Askari Badouei, Babak Khoramian

**Affiliations:** 1 Department of Pathobiology, School of Veterinary Medicine, Ferdowsi University of Mashhad, Mashhad, Iran; 2 Department of Clinical Sciences, School of Veterinary Medicine, Ferdowsi University of Mashhad, Mashhad, Iran

**Keywords:** *Escherichia coli*, Iron, Virulence genes, Typing, Multiplex-polymerase chain reaction

## Abstract

**Background and Objectives:**

*Escherichia coli* is responsible for various enteric and extraintestinal infections in animals and humans. Iron as an essential nutrient, has a proven role in pathogenicity of *E. coli*. Pathogenic *E. coli* benefits of having complicated systems for iron acquisition but our current knowledge is limited because of complexity of these systems. In the present study, three multiplex-PCR assays were developed to screen nine different virulence genes related to diverse iron acquisition systems in *E. coli.*

**Materials and Methods:**

The multiplex-PCR systems were designed and optimized in three panels. Each panel includes a triplex-PCR cocktail. The panels are as follow: panel 1: *iroN, iutA* and *fecA*; panel 2: *fyuA, sitA* and *irp2*; and panel 3: *iucD, chuA* and *tonB*. A total of 39 pathogenic *E. coli* was screened according to the designed multiplex-PCR.

**Results:**

In total, the top three frequent genes were *tonB* (100%), *fecA* (66.6%) and *sitA* (58.9%). With the exception of *fecA* and *tonB*, comparing the prevalence of genes among different origin of isolates (human, cattle, poultry and pigeon) showed significant associations (*P* < 0.05). Moreover, the *iroN, sitA* and *iucD* genes were significantly prevalent (*P* < 0.05) among members of extraintestinal pathogenic *E. coli* in comparison with the group of diarrheagenic *E. coli.*

**Conclusion:**

The current multiplex-PCR assays could be a valuable, rapid and economic tool to investigate diverse iron acquisition systems in *E. coli* for more precise virulence typing of pathogenic or commensal strains.

## INTRODUCTION

*Escherichia coli* is a member of *Enterobacteriaceae* family with a highly heterogeneous population. While the majority of *E. coli* strains are commensal organisms, there are some pathogenic strains which contribute to different infections due to possession of various virulence factors (1). Pathogenic *E. coli* are generally categorized into two main populations which are responsible for enteric infections known as diarrheagenic *E. coli* (DEC) and extraintestinal diseases caused by extraintestinal pathogenic *E. coli* (ExPEC). The DEC group includes various pathotypes such as Enterotoxigenic *E. coli* (ETEC), Enteroaggregative *E. coli* (EAEC), Shiga toxin-producing *E. coli* (STEC), Enteroinvasive *E. coli* (EIEC) and Enteropathogenic *E. coli* (EPEC). The most important members of ExPEC group are Avian pathogenic *E. coli* (APEC), Uropathogenic *E. coli* (UPEC), and Mammary pathogenic *E. coli* (MPEC) which are responsible for extraintestinal diseases in animals or humans (2).

Iron is an essential element for bacteria which is used by different vital cycles and enzymes. Pathogenic bacteria are faced with lack of available iron in their host environment because virtually all of the iron is bound to proteins like lactoferrin, transferrin, ferritin and hemoglobin (3). To overcome the iron limitation, various strategies are applied by pathogenic bacteria in order to gain sufficient amount of iron whether in host or external milieu (4). Since 1968 when Bullen et al., showed the effect of iron in pathogenicity of *E. coli*, the role of iron acquisition systems in pathogenesis has been taken into consideration (5). To date, different systems of iron acquisition have been discovered in *E. coli* which their roles as virulence factors have been studied mostly in Ex-PEC group and less frequently in DEC strains (6–8).

The nine genes that were used for molecular characterization of *E. coli* in the present study included outer membrane receptors for three kinds of siderophores (*iroN, iutA* and *fyuA*), ferric citrate *(fecA)*, ferrous iron *(sitA)* and haem *(chuA)*. Additionally, the genes *iucD, irp2* and *tonB* which contribute in synthesis of aerobactin, yersiniabactin and energy transducer respectively were also included. It should be noted that most iron acquisition systems in *E. coli* are dependent to TonB (4).

Since the ability to acquire iron is related to pathogenicity, genetic profiling of iron acquisition systems can be a valuable asset to screen pathogenic *E. coli* strains. Based on the available literature, most studies targeted only few virulence genes related to iron metabolism in *E. coli* (9, 10). Because of the complexity of iron transport systems, investigating more genes may result in higher resolution pathotyping. Moreover, virulence profiling of *E. coli* based on genes involved in iron acquisition can be applied for DEC, ExPEC and also the emerging hybrid DEC-ExPEC pathotypes (11). Since the complete association of a gene or combination of genes with virulence and establishment of disease in ExPEC strains is not as clear as DEC (12, 13), genetic profiling of *E. coli* based on genes related to iron metabolism can be a valuable aid in clarifying pathogenic and commensal strains and predicting pathogenicity of pathogenic strains.

For molecular virulence typing, different techniques such as: conventional PCR (14), real-time PCR (15) and Whole Genome Sequencing (WGS) through different Next Generation Sequencing (NGS) platforms (16) can be applied. Among the mentioned methods, WGS provides comprehensive data. However, it is an expensive method which is not accessible in many countries at the moment. Real-time PCR assays are time-saving and more cost-effective than WGS, although it is still not available in many laboratories. The methods based on conventional PCR, have the advantages of being the most cost-effective method, which is accessible in most of research laboratories.

In the present study we aimed to develop the first comprehensive multiplex polymerase chain reaction assays to target most important genes related to diverse iron acquisition systems in *E. coli*. The developed method could potentially help researchers to investigate these systems in pathogenic or commensal *E. coli* strains of human and animal origins especially in conditions with no access to new genomic technologies.

## MATERIALS AND METHODS

### Bacterial strains.

A panel of 40 *E. coli* strains was used in the current study. The isolates were randomly chosen from the microbial collection (Ferdowsi University, Mashhad) including APEC (n=8), UPEC (n=10), MPEC (n=10), DEC (n=11) and K12. Other Gram negative bacteria were also tested: *Klebsiella pneumoniae, Salmonella enterica* serovar Enteritidis, *Proteus mirabilis* and *Yersinia enterocolitica*. One clinical strain from each of the aforementioned bacteria was used to ensure specificity and the possible presence of widespread iron acquisition systems in other *Enterobacteriaceae*. All of the strains were identified and confirmed according to the results of standard biochemical tests (17).

### DNA extraction.

A single colony of each bacterial strain was cultured on Luria-Bertani (LB) agar and incubated at 37°C for 18–20 h. DNA was extracted by the boiling method (18). Briefly, a loop of cultured bacteria was added to 500 μl sterile distilled water and was suspended by mild vortexing for 30 s. Then, the suspension was boiled in 98°C for 10 min and was placed on ice for another 10 min. Finally, it was centrifuged (8000 ×g) for 5 min. The supernatant was collected and used as template DNA.

### Simplex PCR design and set up.

The PCR primers for *fecA* and *tonB* were designed using Primer3 based on gene sequences available on the National Center for Biotechnology Information (NCBI) genome databases. The GenBank accession numbers for designing *fecA* and *tonB* primers were NC_000913.3 and CP000468.1, respectively. Other primers were obtained from the available literature (Table 1). Each PCR test was performed in a volume of 20 μl containing: 10 μl *Taq* DNA Polymerase Master Mix RED 2x (Amplicon, Denmark) containing 1.5 mM MgCl_2_, 0.75 μM of each forward and reverse Primers, 5 μl ultrapure water and 300 ng template DNA. PCR products were analyzed by electrophoresis using 1.5% (w/v) agarose gel and Green Viewer safe stain (0.01 v/v).

**Table 1.** PCR conditions and characteristics of the primers used in the study.

**Panel**	**Primer Pair**	**Sequence (5′ to 3′)**	**Function of coded protein**	**T**_**a**_ **Time**	**Primer concentration (μmol)**	**Product Size (bp)**	**Ref**
1	*iroN*	F:AATCCGGCAAAGAGACGAACCGCC	Salmochelin outer membrane receptor	61°C 1 min	0.37	500	(19)
		R:GTTCGGGCAACCCCTGCTTTGACTT					
	*iutA*	F:GGCTGGACATCATGGGAACTGG	Aerobactin outer membrane receptor		0.37	282	(20)
		R:CGTCGGGAACGGGTAGAATCG					
	*fecA*	F:CGGGTATGCGTTTCGAACAT	Ferric citrate outer membrane receptor		0.37	150	Present study
		R:CGAGCCTTCAGTGTTTGCAT					
2	*fyuA*	F: TGATTAACCCCGCGACGGGAA	Yersiniabactin outer	63°C 1 min	0.22	787	(20)
		R: CGCAGTAGGCACGATGTTGTA	membrane receptor				
	*sitA*	F:CGCAGGGGGCACAACTGAT	Ferrous iron outer		0.37	663	(21)
		R:CCCTGTACCAGCGTACTGG	membrane receptor				
	*irp2*	F:AAGGATTCGCTGTTACCGGAC	Contribute in synthesis of		0.37	413	(22)
		R:AACTCCTGATACAGGTGGC	siderophore yersiniabactin				
3	*iucD*	F:ACAAAAAGTTCTATCGCTTCC	Contribute in synthesis of	59°C 1 min	0.37	714	(22)
		R:CCTGATCCAGCTGATGCTC	siderophore aerobactin				
	*chuA*	F:GACGAACCAACGGTCAGGAT	Haem outer membrane		0.37	279	(23)
		R:TGCCGCCAGTACCAAAGACA	receptor				
	*tonB*	F:GCATTGAAGGGCAGGTTAAAGTT	Energy transducer for		0.75	173	Present study
		R:GGATATTCACCACAATCCCACTG	iron uptake systems				

### Multiplex-PCR design and optimization.

Different primers were combined according to their melting temperatures compatibilities to conduct three multiplex-PCR assays and optimized for primer concentrations (Panel 1 to 3). The combination of primer pairs for each panels are as follows: panel 1 consists of three primer pairs for *iroN* (salmochelin siderophore receptor), *iutA* (aerobactin siderophore receptor) and *fecA* (ferric citrate receptor); panel 2 consists of three primer pairs for *fyuA* (yersiniabactin siderophore receptor), *sitA* (ferrous iron/manganese transporter substrate-binding) and *irp2* (biosynthesis of siderophores yersiniabactin); and panel 3 consists of three primer pairs for *iucD* (biosynthesis of the siderophores aerobactin), *chuA* (haem receptor) and *tonB* (energy transducer). The optimum PCR conditions of different panels are listed in Table 1. All PCR tests were performed in a volume of 20 μl containing: 10 μl *Taq* DNA Polymerase Master Mix RED 2x (Amplicon, Denmark) containing 1.5 mM MgCl_2_, various concentration of each Primer, ultrapure water and 300 ng of template DNA. Thermal conditions were as follow: initial denaturation step at 94°C for 3 min, 30 cycles of 94°C for 30 sec, 1 min at different annealing temperatures: 61°C (panel 1), 63°C (panel 2) and 59ºC (panel 3) as mentioned in Table 1 and 72°C for 30 sec. The final extension step was 72°C for 5 min. PCR products were analyzed by electrophoresis using 1.5% (w/v) agarose gel and Green Viewer safe stain (0.01 v/v).

### Sequencing of PCR products of *fecA* and *tonB*.

The DNA products of *fecA* and *tonB* genes were sequenced. To confirm the identity of the amplified regions, sequencing results were analyzed using the nucleotide Basic Local Alignment Search Tool (BLAST) in the NCBI GenBank.

### Statistical analysis.

Data from 39 isolates of pathogenic *E. coli* was analyzed using SPSS software version 16.0. Descriptive statistics and Chi-square analysis were computed in order to determine genes prevalence and clarify any significant association between prevalence of genes and nature of isolates (ExPEC and DEC), origin of isolates (human, cattle, poultry and pigeon) and pathotypes (APEC, UPEC, MPEC, STEC, EPEC, EAEC and EIEC). Statistical significance in this step was assessed at P < 0.05.

## RESULTS

### Multiplex-PCR and sequencing.

**Fig. 1.**
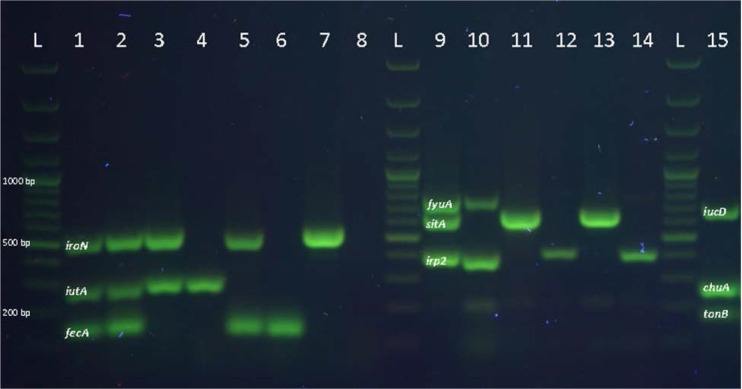
The results of three optimized multiplex PCR and different patterns. L: 100 bp plus ladder, lane 1–8: different patterns for panel 1 *(iroN, iutA, fecA)*; lane 9–14: different patterns for panel 2 *(fyuA, sitA, irp2)*; lane 15: pattern for panel 3 *(iucD, chuA, tonB).*

The results of three optimized multiplex PCR and different patterns are shown in Fig. 1. The sequences of products were confirmed to be parts of *fecA* and *tonB* genes using BLAST in the GenBank (NCBI).

### Genetic profile screening of different *E. coli* pathotypes.

**Fig. 2.**
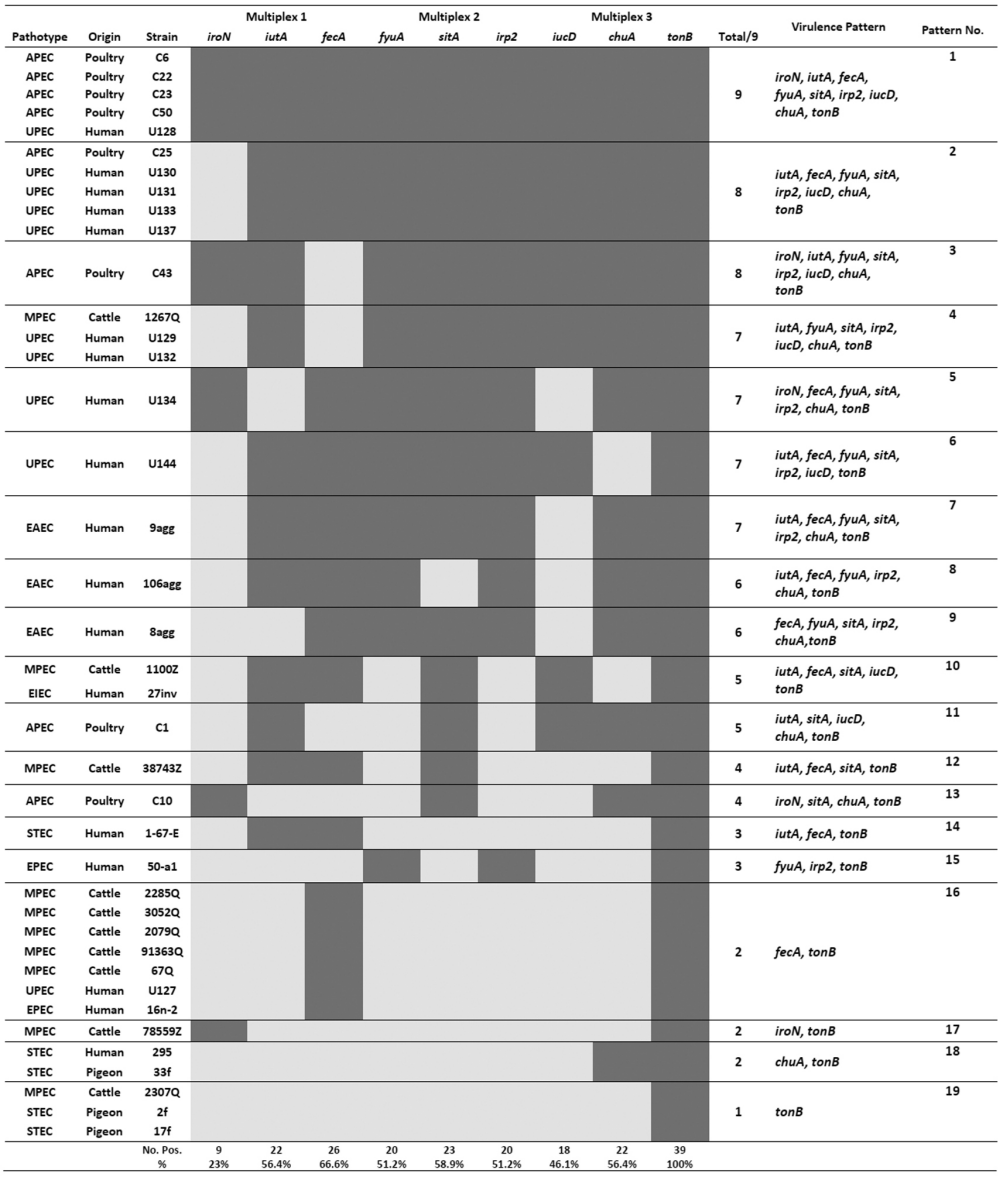
Presence/absence matrix of genes related to gaining iron in *E. coli.* Dark gray indicates presence, light gray indicates absence.

A panel of pathogenic *E. coli* consists of 39 isolates belonging to various pathotypes of Ex-PEC (APEC= 8, UPEC= 10, MPEC= 10) and DEC (STEC= 5, EAEC= 3, EPEC= 2 and EIEC= 1) were investigated. Nineteen different genetic patterns were observed among the tested isolates. The frequencies of investigated genes and different genetic patterns of tested isolates in terms of pathotypes and virulence patterns are summarized in Table 2 and Fig. 2, respectively.

**Table 2.** Frequencies of virulence genes related to iron acquisition in *E. coli* pathotypes.

**Gene**	**Pathotypes**				**Total**

	**APEC N= 8**	**UPEC N= 10**	**MPEC N=10**	**DEC N=11**	
*iroN*	6	2	1	0	9 (23%)
*iutA*	7	8	3	4	22 (56.4%)
*fecA*	5	8	7	6	26 (66.6%)
*fyuA*	6	9	1	4	20 (51.2%)
*sitA*	8	9	3	3	23 (58.9%)
*irp2*	6	9	1	4	20 (51.2%)
*iucD*	7	8	2	1	18 (46.1%)
*chuA*	8	8	1	5	22 (56.4%)
*tonB*	8	10	10	11	39 (100%)

### Genetic profile screening of K_12_ and other members of *Enterobacteriaceae* family.

The K12 *E. coli* strain and *Klebsiella pneumoniae* were detected positive for the presence of *fecA* and *tonB*. Nevertheless, no specific amplicon for the chosen target genes was detected for *Yersinia enterocolitica*, *Salmonella enterica* serovar Enteritidis and *Proteus mirabilis.*

### Statistical analysis.

The *iroN* (P = 0.04), *sitA* (P = 0.027) and *iucD* (P = 0.005) genes were significantly prevalent among members of ExPEC group in comparison with DEC group. Comparing the prevalence of genes among different origin of isolates (human, cattle, poultry and pigeon) revealed significant associations (P < 0.05) for every genes except *fecA* and *tonB* which did not show any significant difference. The same results were obtained when prevalence of the nine genes were investigated through different pathotypes (APEC, UPEC, MPEC, STEC, EPEC, EAEC and EIEC).

## DISCUSSION

The three multiplex-PCR assays designed in the current study efficiently screen nine virulence genes (*iroN, iutA, fecA, fyuA, sitA, irp2, iucD, chuA* and *tonB*) related to different iron acquisition systems in *E. coli*. The chosen genes were shown to have high prevalence, effective role or expected to be important in the pathogenicity of pathogenic *E. coli* in medicine and/or veterinary medicine according to a wide variety of studies (9, 24–27). Most of the previous studies investigated one or only few genes contribute in gaining iron along with other virulence genes or just focused on a defined strategy of iron acquisition like earning iron via siderophores (14, 28, 29); however, the present study represents a practical method to evaluate the genetic potential of *E. coli* in obtaining iron through diverse strategies including three kinds of siderophores (salmochelin, aerobactin and yersiniabactin), ferrous iron, ferric citrate and haem.

According to Table 2, the most frequent detected genes were *tonB* and *fecA*. The gene *tonB* was detected in all the isolates and 66.6% of isolates possessed *fecA.* TonB is responsible for supplying energy for transferring iron and its virulence attribute has been shown in APEC and UPEC (25, 30). Recent studies have mentioned the potential contribution of *fecA* in induction of bovine mastitis (11, 12). In the current study most of MPEC isolates (7/10 isolates) detected positive for *fecA.* Since most efforts to link a gene or set of genes to virulence of MPEC strains were unsuccessful (12), the clarification of the role of *fecA* in mastitis could provide researchers a clue in future studies. Furthermore, most of UPEC isolates (9/10 isolates) had *fyuA, irp2* and *sitA* and all the APEC isolates possessed *sitA, chuA* and *tonB*.

In the present study, the genes involved in iron acquisition via yersiniabactin siderophore ( *fyuA* and *irp2*) were prevalent in APEC isolates which may reflect the key role of yersiniabactin in pathogenesis of APEC strains as other studies have shown (31). Furthermore, in this study, the genes *sitA* and *chuA* were significantly prevalent in isolates recovered from poultry and human. It has been shown that the gene *sitA* contributes to iron acquisition together with *iuc* operon (32) and its presence as a virulence gene has been investigated in numerous studies (33, 34). Moreover, the gene *chuA* is considered a virulence factor not only in DEC but also in ExPEC from various sources (26).

According to the data presented in Fig. 2, in general, the richest genetic profiles belong to APEC and UPEC strains while some of DEC strains have the least number of genes contribute in obtaining iron. Another interesting observation is the wide and scattered distribution of DEC strains among different genetic patterns in contrast to the intensive presence of APEC, UPEC and MPEC strains in some defined virulence patterns. Besides, the most frequent combination of genes was the simultaneous presence of *tonB* and *fecA* which was detected in 66.6% and 71.4% of total pathogenic isolate and ExPEC strains, respectively. It seems that iron acquisition through ferric citrate is one of the basic strategies among members of ExPEC. The multiplex PCRs represented in the current study have an advantage as they include *fecA* and *tonB* in the screening panels for the first time. It should be noted that virulence profiling of iron acquisition genes are only applicable on pure *E. coli* isolates, therefore, determining sensitivity is not a major issue to be addressed for the developed PCR. Additionally, we developed each panel using a moderate number of genes in order to not to sacrifice the sensitivity.

## CONCLUSION

The results of this study showed that current multiplex-PCR assays are applicable as reliable and economic tools which enable researchers to investigate the genetic potential of *E. coli* strains in iron acquisition through diverse systems including siderophores, ferric citrate and haem especially in conditions that application of new genomic technologies is not possible. Furthermore, the multiplex-PCR panels can be used in combination or separately for virulence typing purposes.
